# Adaptive regression modeling of biomarkers of potential harm in a population of U.S. adult cigarette smokers and nonsmokers

**DOI:** 10.1186/1471-2288-10-19

**Published:** 2010-03-16

**Authors:** John H Warner, Qiwei Liang, Mohamadi Sarkar, Paul E Mendes, Hans J Roethig

**Affiliations:** 1 Pharsight Corporation, a Certara Company, Mountain View, CA 94041-1530, USA; 2 Altria Client Services Inc, 601 E Jackson Street, Richmond, VA 23219, USA

## Abstract

**Background:**

This article describes the data mining analysis of a clinical exposure study of 3585 adult smokers and 1077 nonsmokers. The analysis focused on developing models for four biomarkers of potential harm (BOPH): white blood cell count (WBC), 24 h urine 8-epi-prostaglandin F_2α _(EPI8), 24 h urine 11-dehydro-thromboxane B_2 _(DEH11), and high-density lipoprotein cholesterol (HDL).

**Methods:**

Random Forest was used for initial variable selection and Multivariate Adaptive Regression Spline was used for developing the final statistical models

**Results:**

The analysis resulted in the generation of models that predict each of the BOPH as function of selected variables from the smokers and nonsmokers. The statistically significant variables in the models were: platelet count, hemoglobin, C-reactive protein, triglycerides, race and biomarkers of exposure to cigarette smoke for WBC (R-squared = 0.29); creatinine clearance, liver enzymes, weight, vitamin use and biomarkers of exposure for EPI8 (R-squared = 0.41); creatinine clearance, urine creatinine excretion, liver enzymes, use of Non-steroidal antiinflammatory drugs, vitamins and biomarkers of exposure for DEH11 (R-squared = 0.29); and triglycerides, weight, age, sex, alcohol consumption and biomarkers of exposure for HDL (R-squared = 0.39).

**Conclusions:**

Levels of WBC, EPI8, DEH11 and HDL were statistically associated with biomarkers of exposure to cigarette smoking and demographics and life style factors. All of the predictors togather explain 29%-41% of the variability in the BOPH.

## Background

Cigarette smoking is a well known risk factor for cardiovascular diseases [1]. Commonly accepted pathophysiological mechanisms underlying many cigarette smoking associated adverse health effects are inflammation [2,3], oxidative stress [2,4], platelet activation [5,6] and abnormal lipid metabolism [7,8]. Suitable biomarkers of potential harm (BOPH) have been identified for these four different pathophysiological pathways: white blood cell counts (WBC) for inflammation [3,9,10], urine 8-*epi*-prostaglandin F_2α _(EPI8) for oxidative stress [11-13], urine 11-dehydro-thromboxane B2 (DEH11) for platelet activation [11,13,14], and high-density lipoprotein cholesterol (HDL) for abnormal lipid metabolism [15].

The Total Exposure Study (TES) was a stratified, cross-sectional, multi-center study in 3585 adult smokers and 1077 nonsmokers, designed with the primary objective of estimating the exposure to cigarette smoke constituents in a population of U.S. adult cigarette smokers [16]. A secondary objective of the study was to investigate the relationship between cigarette smoke exposure and biomarkers of potential harm. The purpose of this study was to explore relationships between the variables in the TES and four biomarkers of potential harm and to capture those relationships in statistical models.

## Methods

The TES study database contains data on biomarkers of potential harm, biomarkers of exposure (BOE), smoking history, medical history, concomitant medications, clinical laboratory results, and demographics for 3585 adult smokers and 1077 non-smokers. Details about the study have been previously reported [16,17]. The biomarkers of exposure included nicotine equivalents (NICEQ), serum cotinine (COTIN), 4-(methylnitrosamino)-1-(3-pyridyl)-1-butanol (NNAL) and NNAL glucuronides (TOTNN), carboxyhemoglobin (COHb), monohydroxy-butenyl-mercapturic acid (MHBMA), mercapturic acid metabolites dihydroxy-butyl-mercapturic acid (DHBMA), 4-aminobiphenyl (4-ABP) hemoglobin adducts, 1-hydroxypyrene (1-OHP) and 3-hydroxypropylmercapturic acid (3-HPMA). These biomarkers are indicators of exposure to cigarette smoke and represent cigarette smoke constituents. Details about the biomarkers and the smoke constituents represented by these biomarkers can be found in Roethig et al. [16]. They were measured in either urinary samples or blood samples in the TES study [16,17].

### Overview of variables in the data mining database

In the data mining, variables were selected based on their scientific relevance to the targeted biomarkers of potential harm. Table 1 provides an overview of the variables that appear in the data mining datasets and Table 2 has the full names of the biomarkers of potential harm.

**Table 1:** Overview of variable group (and number of variables) that appear in the data mining data set

Group	Variables
BOPH (4)	DEH11, EPI8, HDL, WBC

Special interest variables: BOE (9):	HPMA3, DHBMA, MHBMA, NICEQ, COTIN, OHP, TOTNN, ABP, COHB

Special interest variables: cumulative effects (2)	"AGE", "SMKYRS" (years smoked, excluded from analysis of non-smokers)

Special interest variables: stratification (1)	Smoking Status

Exposure variables (4)	Measures of exposure to exhaust and chemicals, from questionnaire

Exposure variables (non-smokers, 8)	Measures of exposure to secondary smoke, from questionnaire

Exposure variables (smokers, 19)	Measures of exposure to tobacco, including number of cigarettes smoked, tar and nicotine content, presence of menthol, included only in the analysis for smokers

Demographics (13)	Weight, gender, race, geographical location, income, etc.

Vital signs (5)	Respiratory rate, temperature, blood pressure, pulse

Clinical measures (2)	Measures of respiratory capacity, FVC, FEV_1_

General health (20)	General health questions, from questionnaire

Lab values (22)	Clinical chemistry laboratory values

Creatinine clearance (1)	CRCL: 24 h urine creatinine/plasma creatinine

Lab value flags (6)	Lab value flags

Medical history indicators (15)	Medical history findings broken down into 15 categories

Concomitant medications (61)	Concomitant medications broken down into 61 categories

**Table 2:** Variables appearing in the final models, names and abbreviations

Names	Abbreviations	Units/category names
24 h urine 11-Dehydro-thromboxane B_2_	DEH11	ng/24 hr urine
24 h urine 8-*epi-*prostaglandin F_2α_	EPI8	ng/24 hr urine
High density lipoprotein cholesterol	HDL	ng/dL
White Blood Cell Count	WBC	×10^3/uL
24 h urine Nicotine equivalents	NICEQ	mg/24 hr urine
Serum Cotinine	COTIN	ng/mL
24 h urine total 1-hydroxypyrene	OHP	ng/24 hr urine
24 h urine total 4-(methylnitrosamino)-1-(3-pyridyl)-1-butanol (total NNAL)	TOTNN	ng/24 hr urine
Creatinine clearance	CRCL	dL/day
24 h urine creatinine	UCRCAL	mg/24 hr urine
Aspartate aminotransferase	AST	U/L
Alkaline phosphatase	ALKPH	U/L
Serum Triglycerides	TRIG	mg/dL
Platelet count	PLATE	×10^3/uL
High-sensitivity C-reactive protein	CRP	mg/L
Hemoglobin	HGB	g/dL
Age	AGE	Yrs
Weight	WEIGHTK	kg
Vitamin supplements	VITIMINS	yes, no
Alcohol consumption	DRINK	> = once/day, >once/week, once/week, <once/week, no
Non-steroidal antiinflammatory drugs	NSAID	no, yes
Sex	SEX	"female", "male"
Race	RACE	other, native Amer, multi-racial, Caucasian, Asian, black
If greater than	IFGT	
If less than	IFLT	
Equal	EQ	

Separate imputed data mining data sets were constructed for each BOPH. In these data sets, cases were dropped if the value of the dependant variable was missing; values for predictor variables were imputed using methods that are described below. In addition, an unimputed data mining data set was constructed. No cases were dropped from the unimputed data sets and no imputation of missing values was performed on it. The data mining data sets were randomly divided into analysis and validation data sets using an 80/20 split.

### Data mining analyses using random forests

The Random Forest procedure [18-20], a data mining approach for variable selection and model building, was used to perform a preliminary screening of variables for each BOPH (DEH11, EPI8, HDL and WBC). The R statistical package [21] was used for the implementation of Random Forest. Variables that were known to be trivially related to the target BOPH were not included in the initial or subsequent Random Forest runs. In the initial Random Forest analyses, 10,000 trees were generated, and variable importance and cross-validated R-squared statistics were produced. The variable importance effectively ranks all variables in each data set with respect to their ability to predict the target BOPH. At the end, 30 predictor-sets together with cross-validated R-squared statistics were kept. From this list, a large, a medium, and a small predictor set was chosen for input into the Multivariate Adaptive Regression Spline (MARS) procedure [22]. In each case, the large predictor set was chosen to contain 30 variables, while medium and small data sets were chosen to represent natural cut points in the sequence of cross-validated R-squared values.

### Data mining analyses using MARS

Starting with the large, medium and small predictor sets selected by the Random Forests procedure, the MARS algorithm [23] was used to find interpretable models for each BOPH (DEH11, EPI08, HDL, WBC) in each study population (all subjects, non-smokers, smokers). MARS is an innovative and flexible modeling approach that uncovers important data patterns and relationships. It builds flexible models by the use of separate regression slopes in distinct intervals of the predictor variable space. This approach has been increasingly used in recent years in various scientific fields including disease risk research [24,25], human genetics [26] and food sciences [27].

In our study, the large, medium, and small predictor variable sets were augmented by adding all nine BOE and a binary indicator of smoking status. Finally, when ordinal categorical variables appeared in any of the predictor sets, they were replaced with appropriately chosen ordinal dummy variables. Starting with the modified large, medium and small predictor sets, exploratory MARS models were fit for a variety of model types, settings for the number of initial basis functions and penalties for adding new variables.

In order to evaluate the appropriateness of the model, MARS uses generalized cross-validation (GCV) which is residual square errors penalized by a function related to complexity of the model [22]. The numerator in GCV is the average residual squared error and the denominator is a penalty term that reflects model complexity. The use of the denominator is to prohibit selection of a model with many terms that decreases only slightly the residual errors. The GCV statistic is an estimate of the variance for error in a regression model that includes a penalty term for the number of parameters used in the regression. The GCV R-squared statistic is the ordinary R-squared statistic calculated with the variance for error replaced with the GCV statistic [22].

Final MARS runs were performed using exact variable searches (SPEED = 1), three model types (0 = "simple linear model", 1 = "linear model with variable transformations but no interactions", and 2 ="two-way interaction models with variable transformations"), 4 values for the number of initial basis functions (10, 20, 30, and 60), and 2 values for a penalty term for adding new variables (0, and .01). As before, a 10-fold cross-validation was used to estimate the cross-validated (or GCV) R-squared statistic. For each BOPH and predictor set, GCV R-squared, the number of final model parameters, and the number of final model variables were plotted against all other MARS control parameters. These plots (not included in this manuscript) were examined and a preferred model was selected for each BOPH and each study population. Models were preferred if they fit the data well (i.e. had high GCV-R-squared values) and parsimonious (i.e. had few predictors).

Using the imputed analysis data sets the MARS procedure was used to fit models to each of the four BOPH using large, medium, and small predictor sets obtained in Random Forests. This procedure produced closed-form linear regression models that display explanatory power comparable to the best Random Forest models. For each of the four BOPH, a preferred MARS model was selected from the MARS runs based on considerations of goodness of fit and parsimony. The selected models were applied and refit in S-PLUS using the validation data set [28]. Graphical model displays are presented to aid in model interpretation. Applying and refitting models using the validation data set protects against model over-fit that can result from aggressive use of data mining procedures.

Variable importance plots were provided for each BOPH. The marginal R-squared value for a group of predictor variables is the proportion of variance of the dependent variable explained by the group of predictor variables. The delta R-squared for a group of predictor variables is the difference between the marginal R-squared value for the full set of dependent variables and the marginal R-squared value for the full set of predictor variables with the specified group removed. The marginal R-squared is a measure of the quality of prediction obtained from a group of predictor variables acting alone, and the delta R-square is a measure of the improvement in the quality of prediction obtained when a group of predictor variables is added to the other predictor variables in the model.

### Handling of missing data and BLLOQ values

In general, missing data were present in the data set, but rare. In the data mining analysis, it was deemed prudent to impute missing data. Missing continuous variables were imputed in MARS and Random Forest Analyses by replacing missing observations with the median of non-missing cases for each variable: this was done separately for smokers and non-smokers, and for validation and analysis datasets. Imputation was carried out on predictor variables only, cases with missing dependant variables were dropped, not imputed, in the MARS and Random Forest analyses.

Final regression models were fit to data sets in which no imputation was used on any variable, i.e. cases with missing dependant or predictor variables were dropped from the analysis and the number of cases dropped was noted in the regression output. In general, final regression results seemed little changed between imputed and unimputed datasets.

For categorical variables, missing data were handled by adding an additional category as indicated in the following section. This approach does not depend on any assumptions about the sources of missing data and we do not regard it as a form of imputation.

Below the lower limit of quantification (BLLOQ) flags were present in the data set for 9 variables of exposure (NECEQ, MHBMA, TOTNN, DHBMA, OHP, HPMA3, COTIN, COHB, and 4-ABP). If a BLLOQ flag is positive for any of these variables and if the variable itself has a missing value, the value of the affected variable is set to 0. Non-missing value variables BLLOQ values with flags were left unchanged.

### Variable transformations

The Random Forest and the MARS procedure are both designed to find optimal transformations of predictor variables. This minimized the need to perform variable transformations manually. Nonetheless a number of variable transformations were applied to a number of variables. In particular, character variables were transformed to dummy variable. A variable for creatinine clearance was also created. Initial random forest runs found some categorical variables that had natural ordinal interpretations: these variables were replaced with sets of ordinal dummy variables in the MARS and final regression analyses.

### Variable names and naming conventions for the final models

Table 2 presents descriptive names and abbreviated names for all untransformed variables that appear in at least one of the final models. Units (for continuous variable) and category names (for categorical variables), are also provided.

Variable names in final regression models will be of one of the following forms

1. <v.name>

2. <v.name>.EQ.xxx, <v.name>.GT.xxx, <v.name>.LT.xxx, <v.name>.GE.xxx, <v.name>.LE.xxx

3. <v.name>.IFLT.xxx, <v.name>.IFGT.xxx

Here, <v.name> is the short name of a variable from Table 2 and xxx is a number or category selected by MARS. Form 1 is used for variables that enter into the MARS models as linear functions. Form 2 is used to describe dummy variables formed from a categorical variable. In this context "EQ", "GT", "LT", "GE", "LE" stand for "equal", "greater than", "less than", "greater than or equal to", and "less than or equal to". For example, Race.EQ.BLACK designates a variable that is one for blacks and zero otherwise. Alcohol consumption.GT.1 PER WEEK designates a variable that is one for subjects who have more than one drink per week and 0 otherwise. Form 3 is used for transformed continuous variables. For example, CRCL.IFGT.100 (read CRCL if greater than 100) indicates a variable that is equal to CRCL if CRCL is greater than 100 and 100 otherwise. Similarly, CRCL.IFLT.100 is a variable that is equal to CRCL when CRCL is < 100 and equal to 100 otherwise.

Using this notation, regression coefficients can be interpreted as slopes. Positive slopes indicate that the dependent variable increases as the predictor variable increases and negative slopes indicate that the dependant variable decreases as the predictor variable increases. Variables of Form 3 are called transformations of the original variable and are thought of as the result of applying a function (or transformation) to the original variable. Other commonly used transformations that do not appear in our regression output are square root, power, and log transformations.

Collectively, interactions and variables of forms 1, 2, and 3 are called basis functions. The MARS procedure, to be discussed below, may be thought of as a procedure for selecting an optimal set of basis functions that are to be used as the predictor of a given dependent variable. The basis functions used by MARS differ slightly from the basis functions described here. We have chosen to use the basis functions as described here in preference to those used by MARS, because the former seem easier to interpret.

## Results

**Figure 1:**
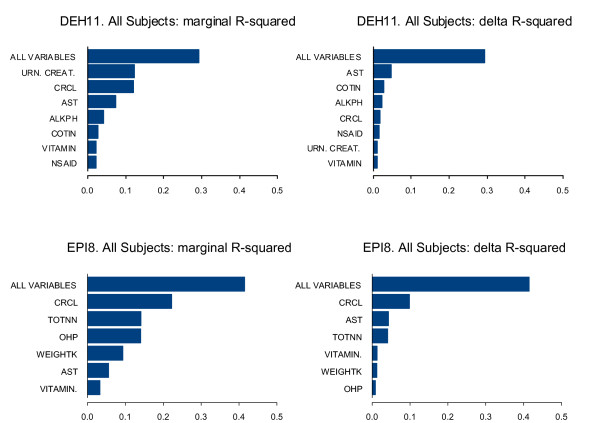
**Variable importance plots for DEH11 and EPI8 for All Subjects (Analysis data set)**.

**Figure 2:**
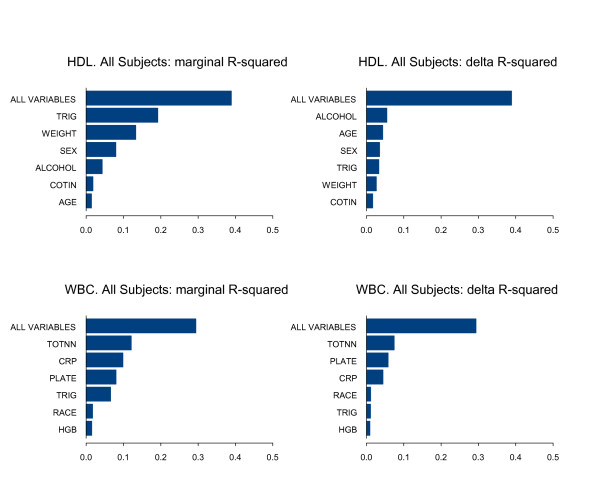
**Variable importance plots for HDL and WBC for All Subjects (Analysis data set)**.

Tables 3 and 4 present parameter estimates, standard errors and p values for the final models for each of the four BOPH for the analysis data set. The parameters often characterize multiple transformations of the same underlying predictor. Figures 1 to 2 show the importance plots of the predictor variables based on Marginal R-squared and Delta R-squared values. All plots were based on unimputed data.

**Table 3:** Parameter estimates in models for DEH11 and EPI8 for all subjects (analysis data set)

a) Model for DEH11	R^**2 **^= 0.29			
**Parameters**	**Value**.	**Std Error**	**t value**	**P value**

Intercept	-4045.3022	752.258	-5.38	<0.001

COTIN.IFGT.11	1.4494	0.123	11.78	<0.001

UCRCAL.IFLT.3036	0.3531	0.0484	7.30	<0.001

CRCL.IFLT.4325	0.3698	0.0391	9.45	<0.001

AST.IFGT.25	17.9064	1.2832	13.95	<0.001

AST.IFGT.126	-18.2428	2.3656	-7.71	<0.001

ALKPH.IFLT.184	3.1360	0.6583	4.76	<0.001

ALKPH.IFGT.184	30.7979	3.6627	8.41	<0.001

NSAID.EQ.YES	-346.0560	38.2528	-9.05	<0.001

VITAMIN.EQ.YES	-211.7183	29.8500	-7.09	<0.001

				

**b) Model for EPI8**	**R^**2 **^= 0.41**			

**Parameters**	**Value**.	**Std Error**	**t value**	**P value**

Intercept	1383.0200	574.483	2.41	0.016

TOTNN.IFLT.57	5.1813	0.7683	6.74	<0.001

TOTNN.IFGT.57	0.5548	0.0593	9.35	<0.001

TOTNN.IFGT.1452	-1.4595	0.3895	-3.75	0.000

OHP.IFLT.473	0.8552	0.1212	7.05	<0.001

CRCL	0.6268	0.0262	23.93	<0.001

AST.IFGT.22	-135.4800	30.5048	-4.44	<0.001

AST.IFGT.24	247.4960	59.5308	4.16	<0.001

AST.IFGT.26	-108.4000	33.1568	-3.27	0.001

AST.IFLT.106	12.2512	2.2425	5.46	<0.001

WEIGHTK	6.2845	0.7274	8.64	<0.001

VITAMIN.EQ.YES	-250.7700	28.4736	-8.81	<0.001

**Table 4:** Parameter estimates in models for WBC and HDL for all subjects (analysis data set)

a) Model for WBC	R^**2 **^= 0.29			
**Parameters**	**Value**	**Std. Error**	**t value**	**P value**

Intercept	-6.4951	1.4436	-4.50	<0.001

TOTNN.IFGT.51	-0.0055	0.0026	-2.17	0.030

TOTNN.IFLT.471	0.0078	0.0023	3.36	0.001

TOTNN.IFGT.471	0.0065	0.0026	2.49	0.013

CRP.IFLT.2	0.3426	0.0548	6.25	<0.001

CRP.IFGT.2	0.0715	0.0096	7.48	<0.001

CRP.IFGT.20	-0.0477	0.0169	-2.82	0.005

PLATE.IFLT.245	0.0106	0.0014	7.69	<0.001

PLATE.IFGT.245	0.007	0.0007	10.64	<0.001

HGB.IFLT.14	0.3242	0.0477	6.80	<0.001

TRIG.IFLT.171	0.0051	0.0008	6.32	<0.001

TRIG.IFGT.503	0.0025	0.0008	3.17	0.002

RACE.EQ.BLACK	-0.679	0.0914	-7.42	<0.001

				

**b) Model for HDL**	**R^**2 **^= 0.39**			

**Parameters**	**Value**	**Std. Error**	**t value**	**P value**

Intercept	55.4292	2.948	18.80	<0.001

COTIN.IFGT.31	-0.0291	0.0036	-7.97	<0.001

COTIN.IFGT.193	0.0258	0.0069	3.75	0.000

TRIG.IFGT.52	-0.5819	0.0786	-7.40	<0.001

TRIG.IFGT.64	0.483	0.0816	5.92	<0.001

TRIG.IFGT.177	0.0753	0.0086	8.76	<0.001

WEIGHT.IFLT.76	-0.267	0.032	-8.34	<0.001

WEIGHT.IFGT.76	-0.0834	0.0157	-5.30	<0.001

AGE.IFLT.55	0.3119	0.0197	15.83	<0.001

SEX.EQ.F	6.72	0.4723	14.23	<0.001

DRINK.EQ.Y	2.0567	0.5099	4.03	0.0001

DRINK.GT.1.PER.WK	7.875	0.5393	14.60	<0.001

The model for DEH11 contains predictors COTIN, UCRCAL, CRCL, AST, ALKPH, NSAID, and VITAMIN (Table 3). The model accounts for 29% of the total variability in DEH11 in all subjects. Higher serum cotinine, urine creatinine and creatinine clearance were predictors of higher 24-hour urinary excretion of 11-dehydrothromboxane B2. Higher serum AST (up to 126 U/L) was mainly a predictor of higher 24-hour urinary excretion of 11-dehydrothromboxane B2 while use of nonsteroidal anti-inflammatory agents and vitamin supplements were predictors of lower 24-hour urinary excretion of 11-dehydrothromboxane B2. In the validation data set the same predictors were significant with the exception of ALKPH.

The model for EPI8 contains predictors TOTNN, OHP, CRCL, AST, WEIGHTK, and VITAMIN and it accounts for 41% of the total variability in EPI8 in all subjects (Table 3). Higher total NNAL (up to 1452 ng/24 h), OHP (if less than 473 ng/24 h), creatinine clearance and weight were predictors of higher 24-hour excretion of urinary 8-*epi*-ProstaglandinF_2α _Type III. Higher serum AST was either a predictor of higher or lower 24-hour excretion of urinary 8-*epi*-ProstaglandinF_2α _Type III, depending on its concentration level. Use of vitamin supplements was a predictor of lower 24-hour excretion of urinary 8-*epi*-ProstaglandinF_2α _Type III. In the validation data set the same predictors were significant with the exception of AST.

The model for WBC in all subjects contains predictors TOTNN, CRP, PLATE, HGB, TRIG, and RACE (Table 4). The model accounts for 29% of the total variability in WBC. Increases in total NNAL (if greater than 51 and less than 471 ng/24 h), high sensitivity c-reactive protein (up to 20 mg/L), platelet count, hemoglobin (if less than 14 g/DL), and triglycerides were predictors of higher WBC count, while race categories of Black and "other" were predictors of lower WBC count. In the validation data set the same predictors were significant.

The model for HDL in all subjects contains predictors COTIN, TRIG, WEIGHTK, HGB, AGE, SEX, and ALCOHOL CONSUMPTION (Table 4). The model accounts for 39% of the total variability in HDL. Higher age, alcohol consumption and female gender were predictors of higher HDL cholesterol, while higher triglycerides was a predictor of lower HDL cholesterol. Higher weight and cotinine (if less than 31 ng/ml) were predictors of lower HDL cholesterol. In the validation data set the same predictors were significant.

Table 5 provides a summary of fit statistics across models for the analysis data set and the validation data set. Figures 1 and 2 gives the importance plots for the predictors for the 4 BOPH.

**Table 5:** Summary of model fit statistics across models for all subjects

Model	Random Forest R^**2**^: Analysis Data Set	MARS GCV R^**2**^: Analysis Data Set	Linear Regression R^**2**^: Analysis Data Set	Linear Regression R^**2**^: Validation Data Set (applied)	Linear Regression R^**2**^: Validation Data Set (Refit)
WBC	0.28	0.27	0.29	0.29	0.31

EPI8	0.40	0.42	0.41	0.35	0.38

DEH11	0.28	0.28	0.29	0.25	0.27

HDL	0.40	0.37	0.39	0.40	0.41

## Discussion

The primary purpose of this analysis was to explore the quantitative associations of biomarkers of exposure and other variables with biomarkers of potential harm related to cigarette smoking. We examined the covariates (or secondary variables) to see whether they could mediate or modulate the effect of biomarkers of exposure. In pursuing this, we developed a systematic approach to the mining of a complex biomarker database. This approach helped us find interpretable linear models that could explain a substantial proportion of the variability for all four of the biomarkers of potential harm (WBC, EPI8, DEH11, HDL). These regression models summarize the information originally contained in 169 variables.

We began by applying a general purpose data mining procedure (Random Forest) to find a list of important variables that could plausibly affect the chosen dependant variables and black box models that relate these variables to the four BOPH. Results from the Random Forest procedure were used as a starting point for the MARS algorithm which produced the multiple regression models. The regression models found by MARS were then applied and refit to a validation data set in SPLUS. It is remarkable that (as shown in Table 5) the R-squared statistics generated by the Random Forests, MARS and regression procedures are in good agreement with each other, suggesting that the proposed models will be reproducible in future studies.

MARS is a nonparametric regression model in which no assumption is made regarding the function relationship between dependent and independent variables [22,29]. Instead of residual plots, other approaches are commonly used for assessing nonparametric regression models [29]. Due to the availability of a relatively large sample size in our dataset, we set aside a validation dataset of 20% of the observations and use it to assess the performance of our models. As presented in Table 5 of our manuscript, the R-squared values of the final models based on the analysis dataset are in good agreement with those based on the validation dataset. This approach confirmed the appropriateness of the models.

The proposed regression models, which involve the transformations of some predictor variables, provide predictive capability comparable to that produced by the Random Forest models. Interactions between dependent variables are not needed. In spite of the generally good agreement of model fit statistics between analysis and validation data sets, the linear models fit to the analysis and validation data sets are not identical, i.e. some variables that are highly significant in the analysis data set do not remain so in the validation data set. These discrepancies, which may suggest aspects of the modeling that will not be generalized to future studies or may be artifacts of the smaller sample size of the validation data set, will be discussed below.

The role of smoking status in our analysis is of special interest. It is notable that smoking status does not appear in any of the regression models. This suggests that the effect of smoking on each biomarker of potential harm is mediated by one or more of the biomarkers of exposure or covariates. 19 additional variables, which measured specifics of smoking behavior, were also entered as candidate variables, but none were included in the final model.

Each model contains at least one BOE predictor: COTIN appears as a predictor of DEH11 and HDL, TOTNN appears as a predictor of EPI8 and WBC, and OHP appears as a predictor of EPI8.

The inclusion of the variables COTIN or TOTNN, instead of smoking status or nicotine equivalents, in the final model for DEH11 or EPI8 does not necessarily mean that these variables by themselves are more biologically important than smoking status or nicotine equivalents. In MARS modeling, the inclusion of a new predictor is dependent on the number of predictors already in the model and the correlation(s) of the new predictor with the remaining predictor(s). COTIN or TOTNN is included in the final model because its contribution and that from other existing predictors accounts for a larger variability in DEH11 and EPI8 than smoking status or nicotine equivalents. On the other hand, the contribution of smoking status or nicotine equivalents is most likely already captured by COTIN or TOTNN and other variables in the model.

As shown in Figure 1, UCRCAL and CRCL are overall important predictors of DEH11, accounting for more variation in DEH11 than the BOE COTIN. Similarly, CRCL is the most important predictor of EPI8, with TOTNN and OHP also playing important roles. The importance of CRCL here is likely related to the fact that DEH11 and EPI8 were both measured in urine. As defined here, CRCL is a marker of kidney function. UCRCAL reflects variability in creatinine production, which is known to be related to muscle mass and is thus influenced by AGE, WEIGHT, and SEX. One of the purposes of this analysis was to investigate various sources of variability, therefore we included the creatinine excretion and creatinine clearance in the model, so that we could understand the magnitude of impact of these factors on the variability. Indeed it was not surprising that, based on the marginal r-squared values (Figure 1), urine creatinine/creatinine clearance had a relatively large contribution on the final models for EPI8 and DEH11. This suggests that normalization for creatinine could potentially reduce the variability in these biomarkers.

The variables TRIG, SEX, WEIGHTK and ALCOHOL CONSUMPTION are all more important predictors of HDL than is the BOE COTIN. The situation is somewhat different for WBC, where the BOE TOTNN appears as the most important predictor, followed by the variables PLATE and CRP.

The model for WBC contains the following predictors: TOTNN, CRP, PLATE, HGB, TRIG, and RACE. The model reflects some well known factors influencing WBC, such as smoking, race and inflammation. The model also indicates a relationship with other hematologic variables such as platelets and red blood cells. Metabolic factors are also known to impact WBC [30].

The model for EPI8 contains the following predictors: TOTNN, OHP, CRCL, AST, WEIGHTK, and VITAMIN. The model suggests that kidney function and body mass are related to the excretion of this biomarker in urine. Exposure to cigarette smoke is thought to increases oxidative stress [11-13], whereas use of vitamin consumption decreases oxidative stress. Oxidative stress may also impact cell membranes, so that enzymes may leak to a higher degree from cells. The weak relationship between AST and EPI8 may be suggestive of the oxidative damage to the cells, since serum AST is localized in heart, brain, skeletal muscle and liver tissue and is generally considered a general biomarker of leaky/damaged cells e.g. hepatocytes as well as myocytes [31]. However this relationship should be interpreted with caution since the contribution of the AST levels to the variability of EPI8 was relatively small (Figure 1). Furthermore AST levels are also reported to be influence by the use of medications [31-33].

The model for DEH11 contains the following predictors: COTIN, UCRCAL, CRCL, AST, ALKPH, NSAID, and VITAMIN. The model suggests that kidney function and muscle mass are related to the excretion of this biomarker in urine. Exposure to cigarette smoke increases platelet activation, whereas use of non-steroidal anti-inflammatory drugs, e.g. aspirin and vitamin consumption decrease platelet activation [11,15]. The relationship with serum enzymes is less clear but might indicate that processes, which activate platelets, impact cell membranes, so that enzymes may leak to a higher degree from cells.

The model for HDL contains COTIN, TRIG, WEIGHTK, AGE, SEX, and ALCOHOL CONSUMPTION. It suggests that bodyweight and male gender impact HDL negatively, whereas older age, female sex and regular alcohol consumption have a positive effect on HDL cholesterol. Higher triglycerides is generally associated with lower HDL cholesterol [34].

## Conclusions

In summary, levels of WBC, EPI8, DEH11 and HDL were statistically significantly related to biomarkers of exposure to cigarette smoking, demographics and life style factors. The statistical models successfully captured a large amount of variability in the biomarkers and depicted the important biological relationships between the biomarker and the effects. Considering the numerous potential sources of variability for the 4 biomarkers and their complex relationships, the R-squared values of 29% to 41% are significant.

## List of abbreviations

The abbreviations of all model variables are listed in Table 2.

## Competing interests

All authors except JHW are or were employees of Philip Morris USA Inc./Altria Client Services Inc. JHW was an employee of Pharsight Corporation when he was conducting the modeling for this paper. Pharsight Corporation received funding from Philip Morris USA for the modeling.

## Authors' contributions

JHW contributed as the primary modeler. He carried out the statistical modeling and participated in developing the manuscript. QL contributed as the corresponding author of the manuscript. He participated in developing the models and finalized the manuscript. MS participated in developing the manuscript. PEM participated in developing the manuscript. HJR initiated the project and participated in developing the models and the manuscript. All authors read and approved the final manuscript.

## Pre-publication history

The pre-publication history for this paper can be accessed here:

http://www.biomedcentral.com/1471-2288/10/19/prepub
